# The clinical and socioeconomic aspects of t-cell receptor excision circle based newborn screening for severe combined immunodeficiency in Southeast and East Asia

**DOI:** 10.3389/fimmu.2025.1549768

**Published:** 2025-05-19

**Authors:** Noelle Yoo, Seongjin Kim, Jane Kim, Jong Gyun Ahn, Insoo Kang, Junghee J. Shin

**Affiliations:** ^1^ Section of Rheumatology, Allergy & Immunology, Department of Internal Medicine, Yale University School of Medicine, New Haven, CT, United States; ^2^ Department of Pediatrics, Severance Children’s Hospital, Yonsei University College of Medicine, Seoul, Republic of Korea; ^3^ Section of Pulmonary, Allergy, Immunology and Sleep Medicine, Department of Pediatrics, Yale University School of Medicine, New Haven, CT, United States

**Keywords:** new born screening, TREC, KREC, SCID, Southeast Asia, East Asia

## Abstract

Severe Combined Immunodeficiency (SCID) is a widely underdiagnosed congenital disease that is fatal by 2-years old if left untreated. Most cases of SCID are diagnosed from the prompting of family history while other cases are sporadic and have no indicators for diagnosis besides the onset of debilitating infections. T-cell Receptor Excision Circle Newborn Screening (TREC NBS) offers an accessible way of flagging for SCID and other T-cell lymphopenia; however, the test implementation rate is low, particularly in Asian countries. This review of the literature will explore the significance of TREC NBS for diagnosing SCID with a focus on the potential impact of widespread implementation on infant healthcare in Southeast and East Asian countries including South Korea, Japan, China, Mongolia, Taiwan, Malaysia, Singapore, and Thailand.

## Introduction

Severe Combined Immunodeficiency (SCID) is a widely underdiagnosed congenital disease that, if untreated, is fatal by age two. The diagnosis of SCID is reliant on prompt screening as the indicators for SCID are typically family history or debilitating infections. T-cell Receptor Excision Circle Newborn Screening (TREC NBS) provides a reliable method for the early detection of SCID and other T-cell lymphopenia. Following the establishment of TREC NBS in several countries, there is growing evidence that SCID is more prevalent than previously estimated. Studies from countries with established TREC screening as well as those with pilot programs have statistically proven the reliability of the test through its high sensitivity and specificity. With low manufacturing and equipment costs, ease of use, and accuracy, TREC screening provides an important and feasible preliminary method for SCID screening in newborns. Factors like consanguinity and genetic isolation raise the risk for a higher prevalence of primary immunodeficiencies including SCID, marking a greater necessity for TREC NBS in many Asian countries (1). TREC screening may have implications in the reduction of infant deaths of unknown cause and Asian newborn health policies. However, more pilot studies and collaborative effort are needed to promote the widespread implementation of TREC NBS.

## Background on T-cell receptor excision circles

T cell progenitors differentiate from stem cells in the bone marrow and migrate to the thymus where they mature into naïve T cells with unique T cell receptors through the T cell receptor (TCR) rearrangement (2). During this process, non-functional pieces of DNA called TRECs are produced (3). TRECs are extrachromosomal episomes that do not duplicate during mitosis and are only found in recently migrated T cells (4). The number of TRECs therefore remains relatively consistent with the number of maturing T cells. Their chemical and quantitative stability long after excision contribute to their success as biomarkers of thymic function and naïve T-cell production (5).

## TREC NBS

Newborn screening through TREC assays provides a minimally invasive, accurate, and convenient method to measure TREC levels in infants. The test is performed through a heel puncture and blood spot collection on special filter papers, known as Guthrie cards. The dried blood spots (DBS) are punched into 96-well filter plates. Rinsing, DNA elution, and qPCR of the samples yield accurate TREC measurements of infants 24 hours and older and provide results within a week (6).

The reliability of TREC NBS can be attributed to multiple factors. In addition to great ease of transportation, DBS are stable so long as they are stored at dry temperatures of -20°C (7). TREC assays can support the demands of entire health systems because of the relatively rapid processing of the samples, durability, transportability, and affordability. The test cost ranges from 4 to 5 USD (8).

While time-tested protocols and commercial kits such as the EnLite Neonatal (Revvity, Waltham, MA, USA) and Spot-it TREC kits (ImmunoIVD, Nacka, Strand, Sweden) provide reliable TREC measurements, challenges arise with the measurement interpretation methods (9, 10). The thresholds for the normal test result have yet to be standardized, however, the consensus seemed to agree on a normal value of TREC as >30–40 copies/μL (11–14). Results below the determined cut-off TREC level were labeled as positive markers for SCID or other forms of T-cell lymphopenia (7). Low to absent TREC levels were flagged as urgent positive results (7). In consideration of the high false positive rate of TREC screening, positive infants may be retested for a confirmation of results and may be monitored with repeat testing (6, 15–17).

As described above, TREC NBS can detect SCID and T-cell lymphopenia, but not B-cell lymphopenia. K-deleting recombination excision circles (KRECs) have been added as a measure in NBS to detect B cell lymphopenia. A study of an Iranian neonatal cohort showed that analyzing both TREC and KREC levels accurately identified both T and B cell lymphopenia (18, 19). It is critical to detect B cell immunodeficiencies including X-linked agammaglobulinemia which usually do not necessarily present with clinical symptoms at birth due to temporary presence of maternally derived Immunoglobulin G. Therefore, implementing KREC testing allows early detection of B cell deficiencies which will ultimately improve the overall health outcomes of affected infants (13, 15, 18, 19). Moreover, incorporating both markers will aid in distinguishing between isolated T cell deficiencies from combined T and B cell deficiencies, facilitating more accurate diagnoses and appropriate management strategies efficiently (13, 15, 18, 19). This comprehensive screening method has been successfully implemented in various studies, demonstrating its efficacy in early detection and intervention for a wider range of immunodeficiencies (13, 15, 18, 19). While challenges related to resource requirements and cost-effectiveness remain, implementation of both TREC and KREC NBS may optimize the sensitivity and specificity of the test by detecting both T and B cell lymphopenia in SCID and ultimately improving the outcomes of neonatal health.

## Severe combined immunodeficiency

SCID is an inborn error of immunity, leading to early death if untreated (20–22). SCID is usually caused by a mutation(s) in genes involved in development, differentiation and/or activation of T, B and/or NK cells including *ADA, RAC2, AK2, IL2RG, Jak3, IL7R, CD3E, PTPRC, CORO1A, NHEJI and DCLRE1C* (23). SCID has an occurrence of 1 in 58,000 infants in the US, with potentially increased frequency in certain populations such as consanguineous or isolated communities (24).

The SCID classifications are typical, leaky/atypical, variant, and Omenn Syndrome (25). Typical SCID and Omenn Syndrome manifest at birth while less acute versions such as atypical/leaky-SCID and variant SCID may present symptoms later in life (25). SCID is usually acquired through autosomal recessive or X-linked inheritance, with a greater frequency in male infants, indicating a higher incidence of X-linked SCID (around 30-40% of all SCID cases) (26, 27).

## Treatment options for SCID

The most common treatment for SCID is a haemopoietic Stem Cell Therapy (HSCT) (28). Long-term success is closely associated with earlier transplantation. In a survey following the outcomes of 177 successful HSCT patients over 38 years, long-term survival rates were highest among those who received treatment before 3.5 months of age (28). Finding a suitable donor is a time-consuming search, during which the SCID patient may continue to experience worsening infections; thus, early diagnosis and initiation of treatment are key to successful HSCT.

In adenosine deaminase deficiency (ADA) SCID, a lack of ADA enzymes from ADA gene mutations impedes the body’s ability to metabolize adenosine and deoxyadenosine waste products that are toxic to lymphoid progenitor cells in the bone marrow and thymus (29). ADA enzyme replacement therapy through weekly intramuscular injections of elapegademase provides a temporary solution before more permanent therapies (30).

Gene therapy extracts stem cells from the patient which are repaired with the insertion of retroviral vectors carrying the normal gene, then replaced into the bone marrow (30). Currently, gene therapy is a viable option for X-linked SCID, ADA-SCID, and Artemis SCID to achieve multilineage integration of modified cells and long-term immune reconstitution (31).

## Status of TREC NBS in Southeast and East Asia

**Figure 1:**
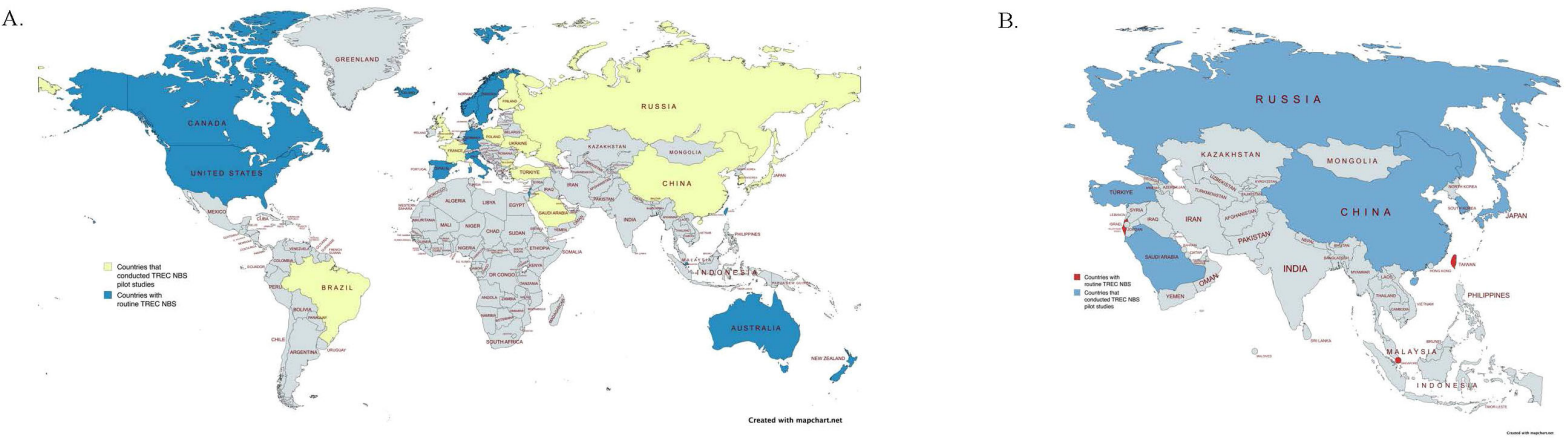
**(A)** Map indicating countries that currently practice routine TREC screening (Canada, the United States, Iceland, Norway, Sweden, Switzerland, Germany, Spain, Italy, Israel, Taiwan, Singapore, Australia, and New Zealand, shown in blue) and countries that have implemented TREC Newborn Screening pilot studies (Brazil, the United Kingdom, France, Finland, Austria, Poland, Ukraine, Bulgaria, Turkey, Saudi Arabia, Russia, China, South Korea, and Japan, shown in yellow) (13, 14, 32–45). Countries without available data or screening programs are shown in gray. This map highlights global efforts to detect SCID in newborns through early screening initiatives. **(B)** Map highlighting Asian countries that have conducted TREC Newborn Screening pilot studies (Russia, Turkey, Saudi Arabia, China, South Korea, and Japan, indicated in blue) and those with established routine TREC Newborn Screening programs (Israel, Singapore, and Taiwan, indicated in red) (14, 32, 33, 43–45). Countries without available data or screening programs are shown in gray.

Currently, China, Taiwan, South Korea, and Japan have performed pilot studies for TREC NBS, with Taiwan and Singapore going on to officially implement nationwide screening for newborns (**Figure 1**). Details of pilot studies in Southeast and East Asia are summarized in **Table 1**.

**Table 1:** Summary of newborn screening pilot studies in Southeast and East Asian countries.

Countries	Period	Duration	Enrolled newborns (n)	Results	
				Typical SCID	Others
China (48)	2010-2011	19 months	106,391	2 cases of typical SCID (IL2RG and RAG1 mutations, respectively)	2 cases of variant SCID 5 cases of 22q11.2 deletion syndrome
Japan (32, 49)	2017-2018	12 months	22,865	Zero cases of typical SCID, likely due to the small sample size of the study. 48 (0.21%) of newborns had positive TREC results	11 cases (23% of infants) of lymphopenia 2 cases 22q11.2 deletion syndrome 1 case of Wiskott-Aldrich syndrome 1 case of a combined immunodeficiency with unidentified causative gene
	2017-2021	52 months	137,484	Two cases of typical SCID (1 in 68,742 newborns)	Six cases of non-SCID PIDs, including: DiGeorge syndrome, 10q11.22–q11.23 microdeletion, 8p inverted duplication/deletion syndrome, cartilage-hair hypoplasia, CHARGE syndrome, and Wiskott-Aldrich syndrome.
Singapore (47)	2019-2020	12 months	35,888	Zero cases of SCID	13 cases of non-SCID T-cell lymphopenia
South Korea (33)	2015	4 months	141	Zero cases of SCID	Zero cases
Taiwan (46)	2010	78 months	920,398	Seven cases of typical SCID, with an incidence of 1 in 131,485 newborns (95% confidence interval, 1/63,693~1/271,434). Hematopoietic stem cell transplantation was performed in six patients before overt infection occurred, and the survival rate was 100%.	136 cases (1 in 6768 newborns) of T cell lymphopenia. Eight cases of SCID variants. 20 cases of 22q11.2 deletion syndrome.

In Taiwan, during the 78 months of screening starting in 2010, seven SCID cases were detected out of 920,398 screened newborns (46). Its effectiveness proven through years of pilot studies, TREC NBS was nationally implemented in Taiwan in 2012 (46). TREC NBS was implemented in Singapore in October, 2019 (47). In its first year of nationwide screening, Singapore had no typical SCID cases found out of 35,888 screened neonates (47). Instead, thirteen cases of leaky SCID and T-cell lymphopenia (TCL) were detected (47). Early detection of TCL can prevent neonates from receiving live vaccines and recommend prophylactic antibiotics for the prevention of opportunistic infections.

A Japanese pilot study screened 80,791 neonates from April 2017 to March 2020 using TREC NBS, and 56,693 neonates from April 2020 to December 2021 using TREC/KREC NBS (32). Of the TREC NBS program sample, six infants were diagnosed with PIDs, zero of which were SCID, and the TREC/KREC NBS program yielded six PID diagnoses, two of which were SCID (32). These infants were able to receive prompt treatment (32). KREC NBS is noted for its high rate of false positives; however, its distinct sensitivity may be useful in early categorization of SCID diagnoses as subjects with T^−^B^−^SCID and Cartilage-hair hypoplasia (CHH) SCID had both abnormal TREC and KREC values while IL2RG-SCID subjects only had abnormal TREC values (32). Although the prevalence of SCID in this study was lower than the estimated average, the TREC NBS and TREC/KREC NBS programs lead to a diagnosis of 55 and 26 non-PID conditions, respectively, demonstrating the expanded benefits of an early newborn screening program (32).

Although South Korea did conduct a TREC NBS pilot study, its findings were limited by a small sample size (**Table 1**) (33). A study with a larger sample size assessing primary immunodeficiency (PID) cases across 27 hospitals in South Korea from 2001–2005 recorded 152 cases, concluding a PID prevalence of 11.25 patients per million children in South Korea (50). Four (2.6%) of these cases were SCID patients and a total of 27 (17.8%) of PID patients had T-cell deficiencies or combined immunodeficiencies, suggesting the potential benefit of TREC NBS in South Korea (50).

Presently, there have not been any TREC NBS pilot studies performed in Mongolia, Macau, Brunei, Cambodia, Indonesia, Laos, Myanmar, the Philippines, Thailand, Timor-Leste, and Malaysia (51). The status of TREC NBS initiatives in North Korea remains unknown (51). Although these countries have not executed TREC NBS pilot studies, there are some SCID case studies of interest and reported cases that indicate a relevance for a screening program. In a review of all published PID cases in Malaysia from 1979 to 2020, of the 119 PID cases, the most common diagnosis was SCID, with a total of 22 patients. All 8 of the SCID patients who had received HSCT were confirmed to be alive (52). Of 65 cases of PIDs spanning 18 years in a Thailand hospital, SCID comprised 12 (17.9%) of the patients and there was a total of 17 (25.4%) combined T cell and B cell immunodeficiency cases (53). The lowest rate of survival was found in the SCID patients, with a mortality rate of 52.9% (53). Three of the SCID patients had Bacillus Calmette-Guérin (BCG) infections from the BCG vaccine despite a contraindication in family history (53). Establishment of a screening program would delineate stricter standards for live vaccinations and deter preventable complications.

## Comparative analysis of global implementation of TREC NBS

In North America, particularly in the United States, the inclusion of SCID in the Recommended Uniform Screening Panel (RUSP) has been pivotal in nationwide implementation. The existence of centralized public health policies, extensive healthcare infrastructure, and robust governmental funding has enabled comprehensive integration of TREC NBS programs across states, ensuring equitable access and standardized protocols (11).

Europe has adopted a similar structured approach, with centralized public health directives facilitating uniform screening practices. For instance, Switzerland and Spain’s nationwide screening programs benefited significantly from clear policy guidance and government support, resulting in high implementation coverage and early identification rates of SCID cases (54).

Contrastingly, Southeast and East Asian countries exhibit considerable variability in their approaches to TREC NBS implementation due to differences in healthcare governance, resource allocation, and infrastructure. Countries such as Taiwan and Singapore successfully implemented nationwide programs through centralized healthcare systems, proactive governmental initiatives, and sufficient economic resources (46, 47).

However, countries with decentralized or fragmented healthcare governance, such as Thailand and Vietnam, face substantial hurdles in establishing nationwide screening (55). Limited budgets, insufficient specialized healthcare professionals, and inadequate laboratory infrastructure hinder effective program implementation, necessitating targeted strategies to overcome these systemic barriers (56).

In Western regions, the strategic integration of SCID screening into existing newborn screening programs for metabolic and genetic disorders has been advantageous, allowing for rapid adoption and streamlined processes (57). Economic analyses have consistently shown cost-effectiveness in these regions, promoting political support and public acceptance (34, 57).

Conversely, in Asian regions, particularly in lower-income settings, strategic planning must account for economic constraints and cultural attitudes towards genetic conditions. Initiatives in Malaysia illustrate potential pathways, suggesting integration with existing newborn screening programs to optimize resources, demonstrating the need for context-specific strategies to achieve successful outcomes (58).

The differences in policy frameworks and implementation strategies between Southeast and East Asia and non-Asian regions highlight the necessity for tailored approaches. Effective implementation of TREC NBS globally requires an understanding of regional public health policies, economic conditions, and cultural contexts to ensure successful and sustainable outcomes across diverse healthcare environments.

## Clinical importance of TREC NBS in Southeast and East Asia

Southeast and East Asia are known to have a high and intermediate rate of neonatal mortality, respectively (59). In Southeast Asia, the under-5 mortality rate was 32 deaths per 1000 live births, and neonatal mortality rate was 20 deaths per 100 live births in 2019 (60). About 52% of under-5 mortality is attributed to neonatal deaths and the leading causes are complications of prematurity, followed by pneumonia and diarrhea (60). Although social factors including poor nutrition status, inadequate water, sanitation, and hygiene conditions make them susceptible to infections, we cannot rule out SCID as the cause of neonatal death.

PIDs are more likely to be detected in countries that have controlled the rates of deaths from common infections so that children with PIDs survive long enough to be identified (61). It has thus been determined that PIDs are more likely to go undetected in countries where the under-5 mortality is higher than 15 out of 1000 live births (61). Vietnam has an under-5 mortality of 20, Timor-Leste: 49, the Philippines: 28, Myanmar: 40, Indonesia: 21, and Cambodia: 24 (62). With under-5 mortalities higher than 15, these countries likely have a significantly greater underdiagnosis rate of PIDs, including SCID, that could be compensated for by TREC NBS initiatives.

Diagnosis of SCID requires a significant family history to prompt screening. However, over 80% of SCID cases are sporadic, having no prior indications other than clinical symptoms (63). In these cases, diagnoses are made only after the infant has experienced debilitating infections that suggest SCID, or even death (64). Therefore, initiating pilot studies and establishing TREC NBS in Southeast and East Asia may prevent life-threatening infections in neonates as nations around the world including the United States, Canada, Singapore, Taiwan, and most European regions appreciate the clinical benefits of pilot studies and now fully implement TREC NBS as one of their official newborn screening cohorts (**Figure 1**).

Pilot studies with substantial sample sizes have demonstrated the authentic effect of TREC NBS and its functionality as newborn screening for SCID. As one example, out of the 130,903 screened in the first two years of implementation of TREC NBS in Spain since 2017, 30 SCID patients were successfully diagnosed and treated (35). In the United States, 52 SCID cases were detected through TREC NBS out of 3,030,083 neonates screened from 2008 to 2013 (11). By the end of 2018, all states implemented TREC-NBS as part of their universal newborn screenings (11). As a result, as of 2024, SCID disease is one of 38 core conditions on the Recommended Uniform Screening Panel (11). The number of SCID cases detected through TREC NBS implies the benefits given to families of neonates who were unaware of SCID or could not recognize the need to get their children screened. The neonates who were diagnosed with SCID disease after screening would immediately get treated, possibly preventing life-threatening infections.

The practicality of TREC NBS in detecting the SCID patients can be also analyzed by the change in incidence rate before and after implementation. For example, according to research conducted in University of Zurich, after the nationwide implementation of TREC NBS in Switzerland in 2019, the incidence of SCID disease was reported at approximately 1 in 25,000 infants for the first two years compared to that in the previous years which was approximately 1 in 66,000 infants (65). This result supports the hypothesis that a number of SCID cases can be underestimated without an application of universal newborn screening and that TREC NBS would give an alert to potential SCID patients who are not yet diagnosed.

In parallel, according to the first multistate report of TREC NBS for SCID in the United States, the newly observed incidence of SCID was 1 in 58,000 births, but estimated incidence based solely on clinical diagnoses before newborn screening was 1 in 100,000, which was lower than that after the implementation of newborn screening (11). Higher incidence rate again implies the effectiveness of TREC NBS in detecting potential SCID patients.

Effective and prompt diagnosis of SCID has a significant association with the survival rate. Early diagnosis is critical to improving subsequent treatment outcomes, with significant contributions to infant survival rate. In a study analyzing the correlation of TREC NBS and survival rate, there was a statistically significant difference in the survival rates of newborns not tested at birth (58%) and those tested at birth (64). An independent study showed that the treatment of SCID before infections had a 90% survival rate while treatment after infections resulted in only a 50% survival rate. Pai et al. also reported that survival rates after transplantation were 82% in infants that had prior infections, 90% in older infants with no prior infections, and 94% in infants that had received transplants at 3.5 months old or younger (66). Survival likelihood increases by around 8% with an avoidance of infection, with an additional increase of 4% with earlier treatment. Early diagnosis is therefore crucial in promptly informing infant caretakers of infection risks and to seek earlier treatment. Heimall et al. uncovered a survival rate of 90% in infection-free infants that decreased to 81% in patients with an active infection (67). Infections that may cause irreversible organ damage, reduce treatment efficacy, or render an infant too weakened for treatment can be avoided with earlier prognosis and care. With the continued research and development of gene therapy and HSCT currently taking place, paired efforts to implement nationwide TREC screening programs will only continue to improve survival outcomes and infant healthcare.

In the study by Heimall et al. surveying the mortality rates of 158 SCID infants, 61 infants were reported to have died (67). 51% of the 61 deaths were infants who had been diagnosed and treated, 20% had only been diagnosed, and 29% were diagnosed only after death (67). The study found that the biggest barrier to treatment was not a lack of suitable donors, financial restrictions, or lack of access to facilities. The largest barriers to treatment were that the infants had already died (59% of untreated infants) or were too ill to receive treatment by the time they were diagnosed (29% of untreated infants) (67). Early diagnosis plays a crucial role in SCID infant survival, demonstrating the need for systematic TREC NBS. Heimall et al. also found that *Pneumocystis* and *Candida* infections were less frequent amongst SCID patients diagnosed by TREC NBS or a positive family history versus SCID patients diagnosed by the onset of persistent infections, indicating that earlier diagnosis through NBS is successful in preventing certain infections (67).

Therefore, given the crucial role of TREC NBS for early diagnosis of SCID leading to prompt and effective treatments for infections of neonates, implementing TREC NBS may decrease neonatal mortality in Southeast and East Asia. However, the sensitivity and specificity of TREC NBS should be carefully evaluated through pilot studies, because missing cases due to false negative results defeats the purpose of this screening test (64). The diagnostic sensitivity of TREC NBS makes it a powerful tool for early SCID detection, but the downstream effects of false-positive results also warrant consideration. While these tests are necessary to ensure an accurate diagnosis, they can introduce both financial costs for additional confirmatory testing including flow cytometry and the potential for extended patient monitoring which may strain healthcare resources and may strain families financially and emotionally. In high-income countries, the follow-up cost per false-positive can range from $100 to over $700 USD depending on the clinical pathway (7). In lower-resource settings, these expenses may be more burdensome where out-of-pocket healthcare costs are high. While it may not be realistic to earmark separate funding for false-positive cases, policymakers and program designers should account for these costs in overall budget planning. Minimizing unnecessary downstream testing through standardized retesting protocols, physician training, and public education may be a more feasible and cost-conscious approach in national TREC NBS rollouts. Moreover, receiving a false positive result can cause significant emotional distress for parents. Despite subsequent confirmatory tests showing that the child is healthy the initial uncertainty and the possibility of a Severe Combined Immunodeficiency diagnosis can cause considerable anxiety and stress for families. A review manuscript highlighted that almost 10% of parents with false-positive NBS results reported clinically significant stress and ongoing concerns about their child’s health and future (68). Similarly, a study in China found that mothers of infants with false-positive results experienced higher stress levels and were more likely to perceive their child as vulnerable, leading to increased parental care and more frequent hospitalizations (69). Therefore, establishing a TREC NBS protocol with improved accuracy and reduced false positive rates is crucial to reduce unnecessary economic burdens on healthcare systems and minimize anxiety and stress for parents and healthcare workers.

## Socioeconomic importance of TREC NBS in Southeast and East Asia

The economic, geographic, and cultural differences in Southeast and East Asia add to the challenges but also importance of establishing newborn screening systems.

Implementation of Newborn dried bloodspot screening (NDBS) in Asia has been slow primarily for economic reasons (56, 70). However, early diagnosis has tremendous effects on the cost difference of SCID treatment. A U.S.-based study by Ding et al. estimated an average hospital care cost of 100,000 USD for infants treated before 3.5 months and 450,000 USD for infants treated after 3.5 months–a 350,000 USD increase avoidable with prompt diagnosis and treatment (8). Therefore, though the exact numbers are unknown, implementation of TREC NBS may cost-effectively improve neonatal mortality in Southeast and East Asia. However, we acknowledge that the cost of TREC NBS is based on Western countries and the economic effect of the cost in Southeast and East Asian countries is unclear.

The economic burden of not implementing TREC NBS extends far beyond the direct cost of delayed SCID treatment. From a systems-level perspective, every missed SCID diagnosis represents a lost opportunity for cost containment, resource efficiency, and life-years saved (71). Early identification through newborn screening enables curative treatment before infections develop, often at dramatically lower cost. But it also avoids downstream healthcare expenditures such as prolonged neonatal ICU stays, specialist referrals, chronic immune support, and end-of-life care—none of which have been systematically quantified in many Southeast and East Asian countries (72).

Moreover, late diagnosis introduces inefficiencies into public healthcare delivery. Infants with undiagnosed SCID may present repeatedly with vague or severe infections, often leading to multiple misdiagnoses and inappropriate treatments. This cycle not only endangers the patient but misallocates scarce healthcare resources—particularly in low- and middle-income countries where pediatric immunologists and tertiary care centers are limited (52, 73). Implementing TREC NBS allows for earlier triaging, potentially reducing the strain on overwhelmed referral systems and enabling better distribution of care.

Failing to screen for SCID can have intergenerational economic impacts, especially when families withdraw from the workforce to provide care, experience child loss, or face long-term medical debt. In regions with rapidly aging populations and declining fertility—such as Japan, South Korea, and Singapore—investing in infant health through programs like TREC NBS is not just clinically prudent but demographically strategic (55). Healthy childhood cohorts reduce future dependency ratios and help sustain national productivity (74).

The absence of TREC NBS highlights a missed opportunity to strengthen preventive health infrastructure, which has been shown to be more cost-effective than reactive approaches to treatment. Frameworks such as cost per disability-adjusted life year (DALY) averted or incremental cost-effectiveness ratios (ICERs) could help policymakers evaluate the value of screening programs relative to competing health priorities (75). These models have been successfully applied to vaccines and metabolic disease screening programs in Asia, but rarely to SCID, highlighting a need for tailored pilot study designs that incorporate longitudinal cost-benefit analysis (76–78).

Establishing financial plans for TREC NBS in each country in Southeast and East Asia will provide a better understanding of economic burden of implementing TREC NBS in East and Southeast Asia. Financial planning should consider infrastructure costs including laboratory equipment (e.g. PCR machines and reagents), training and workforce development, data management systems and establishing NBS centers where follow-up diagnostic testing, treatment and follow up plans can be facilitated. These upfront costs should be balanced with the long-term savings achieved through early detection and intervention of immune deficiencies. Pilot programs can be conducted in countries within Southeast and East Asia to develop a financial model account for infrastructure costs, staff salaries, training programs and follow-up testing. Collaborative effort with governmental agencies, public health officials, healthcare providers should be explored for funding options and identifying potential cost-sharing models throughout Countries in Southeast and East Asia.

Many Southeast and East Asian countries, such as Mongolia, China, South Korea, Japan, Taiwan, Singapore, Malaysia, and the Philippines, face geographic isolation due to natural and man-made barriers, including deserts, mountain ranges, coastlines, and political borders (79). The genetic variation of populations from such geographically isolated countries may be influenced by a founder effect and contribute to the consequent prevalence of congenital disorders such as SCID (79–81).

Consanguinity, which increases homozygosity and the likelihood of monogenic disorders, is present in 23.2–31.9% of marriages in Malaysia depending on region, 17.8% of marriages in parts of Indonesia, 0.3–4% of marriages in Singapore, 18.9–29.8% of marriages in some regions of Thailand, 3.9% of marriages in Japan, and 2% in China (82–85). These consanguinity rates are significantly higher than or are comparative to rates in countries with established TREC NBS, demonstrating a potentially higher SCID prevalence and greater role for TREC NBS in many Asian countries. The US has a rate of 0.1%, Israel has a rate of 10.4%, and the TREC NBS countries in Eastern Europe have a range of 0.1%–2.6% (86). However, the rates of consanguinity in some Southeast and East Asian countries remain unknown suggesting that pilot studies of TREC NBS in these countries will guide us to assess the frequency of SCID in these regions.

While consanguinity is not uniformly high across all of Asia, its documented presence in certain Southeast and East Asian regions should be considered in the broader context of SCID screening implementation. Although current data do not support a generalized claim that SCID is more prevalent in Asia overall, consanguinity is a well-established risk factor for autosomal recessive primary immunodeficiencies, including SCID (1). Therefore, tailoring screening policies to local genetic, demographic, and public health contexts—including known risk factors like consanguineous marriage patterns in certain provinces or ethnic groups will be critical for implementing TREC NBS in Southeast and East Asia.

As mentioned above, the incidence rate of SCID in Switzerland increased from approximately 1 in 66,000 infants to approximately 1 in 25,000 infants for the first two years with implementation of TREC NBS (65). Therefore given the critical clinical and socioeconomic factors in Southeast and East Asia, implementation of TREC NBS will increase early diagnosis of SCID in these regions.

Current socioeconomic analyses surrounding TREC NBS implementation address general considerations for budget planning and highlight the potential economic benefits of early SCID detection. However, comprehensive region-specific hospital cost data from Southeast and East Asian countries remain limited. Although existing economic evaluations from higher-income regions provide useful guidance, further research into localized hospital costs and healthcare expenditures within Asian contexts would offer greater precision in assessing the financial feasibility of nationwide TREC NBS programs. Enhancing the availability of region-specific economic data could better inform targeted resource allocation and facilitate more tailored approaches to implementing sustainable NBS programs throughout Asia.

## Potential approaches to establish TREC NBS program in Southeast and East Asia

In an expansion of existing NBS using Drop Blood Screening (DBS), Kumarasamy et al. performed a feasibility study of a nationwide TREC NBS program in Malaysia by assessing cost-effectiveness, accessibility, and necessity (87). In addition to a favorable long-term infant healthcare and economic outlook, the study also suggested a possible inlet for TREC NBS: an expansion of the country’s existing newborn screening using DBS including various metabolic syndromes. This approach of building on current NBS protocols has potential in countries such as Laos, Thailand, China, Japan, South Korea and the Philippines, which regularly screen newborns for inborn errors of metabolism (88–90).

The cost-effectiveness of TREC NBS has been demonstrated in high-income countries but remains a critical concern for middle- and low-income countries in Southeast and East Asia. The cost of infrastructure—including purchasing equipment, training personnel, and implementing secondary screening methods—can be prohibitive. However, as Malaysia’s feasibility study suggested, there is potential for cost-effective integration by building on existing newborn screening programs, particularly those already screening for metabolic disorders (87). Collaborative efforts with international organizations like the Asia Pacific Society for Human Genetics could help facilitate the sharing of resources, funding models, and expertise, providing a feasible pathway for implementation.

It is notable that Singapore and Taiwan, two countries with highly ranked universal healthcare systems, have established nationwide TREC NBS (67). Countries with similar healthcare systems and national wealth such as South Korea and Japan may refer to these existing TREC NBS programs to model their own initiatives (67). Asian countries with decentralized healthcare systems may find guidance from pilot studies in pursuing a more localized approach.

## Establishing TREC cut-off value for TREC NBS in Southeast and East Asia

General screen algorithms for TREC NBS are similar among screening sites, however, TREC cut-off values and handling results vary along different screening sites. A systemic review manuscript on TREC NBS suggested that using a TREC cut-off value of maximal 25 TRECs/μl and incorporating the collection of a repeat Dried Blood Spot (DBS) from patients with an abnormal screening result in the screening algorithm would be most effective in screening SCID (15). TREC assay result was also reported as multiple of the median (MoM) rather than using conventional copy numbers. MoM is a measure of how far an individual test result deviates from the median (91). Therefore, it allows normalization of TREC assay data from different laboratories, so that individual test results can be compared regardless of the method, assay, or reagents used (91). However, if MoM value is used to define the positive screening cutoff, it is necessary to monitor the population median (91). The previously done pilot studies in Tiwan, Singapore, Japan and China used different cut-off as 25, 18, 31 and 40 copies/μl, respectively (32, 46–49). Therefore, additional pilot studies to establish the population median of TREC values may allow us to use MoM values to report TREC assay results in a unified way in Southeast and East Asia. To promote harmonization of TREC NBS testing protocol in Southeast and East Asia will require establishing a regional collaborative network for standardizing quality assessments and development of unified guidelines and protocols. Working Group of the Asia Pacific Society for Human Genetics on Consolidating Newborn Screening Efforts in the Asia Pacific Region has been established to facilitate formation of the Asia Pacific Newborn Screening Collaboratives (56). Collaboration with the working groups of the Asia Pacific Society for Human Genetics on Consolidating Newborn Screening will be a critical step toward harmonizing TREC NBS testing protocol.

## Socioeconomic factors to consider for establishing TREC NBS in Southeast and East Asia

Factors including neonatal mortality, infection burden in neonates and consanguinity rate in the countries should be considered as critical reasons for implementing TREC NBS. However, given the economic burdens on healthcare systems and increased anxiety and stress for parents due to the false positive results of TREC NBS, development of accurate and efficient TREC NBS protocol is crucial. Therefore, collaborative effort with the working groups of the Asia Pacific Society for Human Genetics in consolidating newborn screening supported by the U.S. National Institutes of Health, the Centers for Disease Control and Preventing, the U.S. National Institutes of Health, the Centers for Disease Control and Preventions, and U.S. National Newborn Screening and Genetics Resource Center, the international Society for Neonatal Screening will be necessary (56). Furthermore, implementation of TREC NBS encompasses multiple disciplinaries not only to diagnose SCID but also to plan for prompt and effective treatment including Hematopoietic Stem Cell Transplantation (92). Therefore, a multidisciplinary collaborative effort of specialists including but not limited to immunologist, hematology/oncologist and infectious disease specialist is critical to properly diagnose and treat patients with SCID with implementation of TREC NBS. As of 2019, Malaysia had a total of 5 clinical immunologists due to immunology not being recognized as an official subspecialty (93). Similarly, Vietnam only has 5 immunologists/allergists per 100 million population, the Philippines 5 per 10 million, Hong Kong 4 per 10 million, and Indonesia 2 per 10 million (73). An insufficiency in manpower coupled with conflicting economic and social priorities led to an underdiagnosis in PIDs in Malaysia (93). Medical education, such as awareness of PIDs, has an inverse relationship with national wealth (55). TREC NBS initiatives could raise awareness of PIDs, particularly fatal diseases like SCID, in Asian countries or regions with lower rates of medical education. It could also potentially compensate for the lack of manpower found in Southeast and East Asian countries by shifting the burden of medical awareness and personal healthcare initiatives from the patient and few physicians, placing it on a routine procedure established by a joint effort of international organizations, governmental agencies, and public health officials of countries in Southeast and East Asia.

The demand for better healthcare is rising across many Asian regions due to factors such as increasing education levels, aging populations, heightened awareness of human rights, and rapid urbanization. Decreasing fertility rates and aging populations are a concern for multiple Asian countries such as South Korea, Japan, Singapore, and Thailand (55). Improving infant healthcare through TREC NBS may address the challenges of an aging population and concerns that may affect people’s willingness to have children. As these socioeconomic trends shift the focus toward the need for better medical education and higher-quality care, TREC NBS initiatives are poised to both support and be supported by these broader efforts.

## Conclusion

The implementation of TREC NBS across Southeast and East Asia holds the potential to reduce infant mortality by enabling early detection and treatment of SCID and other T-lymphopenia disorders. Addition of KREC to the TREC NBS may further improve the sensitivity and specificity of detecting both T and B cell lymphopenia in SCID. The success of established screening programs globally, along with promising results from regional pilot studies, illustrates the importance, feasibility, and cost-effectiveness of expanding screening efforts nationwide. In Southeast and East Asia, challenges such as resource allocation, infrastructure development, and improved standardization remain to be resolved. Therefore, collaborative efforts across countries with the Asia Pacific Society for Human Genetics in consolidating newborn screening could streamline the adoption of TREC NBS, ultimately reducing SCID-related fatalities and improving healthcare for future generations.
